# Finite Element Assessment of a Novel Patient-Specific Mandibular Implant for Severely Atrophic Ridge

**DOI:** 10.1155/2024/9735427

**Published:** 2024-08-29

**Authors:** Alireza Parhiz, Reza Nourishirazi, Amirali Asadi, Morad Karimpour

**Affiliations:** ^1^ Department of Maxillofacial Surgery School of Dentistry Tehran University of Medical Sciences, Tehran, Iran; ^2^ School of Mechanical Engineering College of Engineering University of Tehran, Tehran, Iran

**Keywords:** 3D printing, atrophic mandible, dental reconstruction, patient-specific, subperiosteal implant

## Abstract

**Purpose:** Dental reconstruction for patients diagnosed with severe mandibular bone atrophy using common dental implants is a challenging process. In such cases, surgeons may encounter challenges such as insufficient available bone, soft tissue, damage to the inferior alveolar nerve, and even the risk of bone fracture. In this study, a new design concept of mandibular patient-specific implants for severely atrophic ridges followed by finite element evaluation was presented to investigate the mechanical functionality of the concept.

**Method:** The implant is comprised of two modular parts including an inferior border cover and a horseshoe-shaped structure. This horseshoe segment fits into the cover and is then screwed to it using two screws on each side. A 1 mm deflection was applied to a reference point located between the two anterior posts to extract the resulting Von Mises stress distribution in each part and the reaction force on the reference point which corresponds to the chewing force that the patient must apply to deform the horseshoe. This 1 mm gap is a design consideration and critical distance that horseshoe contacts the gingiva and disturbs the alveolar nerve.

**Results:** The results revealed that load was transmitted from the horseshoe to the cover, and there were no stress contours on the body of the mandible. However, stress concentration was observed in screw locations in the mandible, the amount of which was decreased by increasing the number of used screws. In horseshoe, stress concentration values were around 350 MPa, and the measured reaction force on the reference point was just under 200 N.

**Conclusion:** The finite element analysis results showed that this concept would be functional as the minimum load would be transmitted to the mandibular ridge, and since the patients diagnosed with atrophic ridge are not able to apply load to an amount near 200 N, the horseshoe would not contact the gingiva. Also, it is concluded that increasing the number of bone screw fixations would decrease the risk of long-term screw loosening.

## 1. Introduction

Edentulism leading to vertical bone resorption is a challenging problem in both mandibular and maxillary residual ridges. Generally, oral rehabilitation with conventional dental implants is impossible due to insufficient bone quality, volume, or height, which is the leading cause of bone fracture and implant failure [1, 2]. Conventional surgical procedures like mandibular onlay bone grafting, where the donor block graft is placed and screwed in a vertical-buccal direction, the sandwich technique for vertical bone augmentation, nerve transposition, or relocation in the posterior mandible, all-on 4, all-on 6, and overdenture are used to rehabilitate the occlusion in the atrophic mandible [3–5]. But in the severely atrophic mandible (Type 3 Division D Misch and Judy classification), patients are usually diagnosed with inadequate interarch space to accept the implants and overdentures. Also, bone grafts may not result in sufficient strength due to poor integration; thus, none of these techniques may be applicable most of the time.

Using customized 3D-printed subperiosteal implants and ramus frames are alternatives to overcome this issue, providing structures to transmit the occlusal load from the abutment or post to the bone [6, 7]. However, this method would be useless in patients with severe bone loss as it increases the risk of mandibular fracture and failure. On the other hand, in patients diagnosed with mandibular atrophy, the superficial location of the inferior alveolar nerve may interfere with the placement of subperiosteal implants and screws [8].

This research is aimed at providing a new design concept for patient-specific mandibular implants for patients diagnosed with severely atrophic mandible, transferring the minimum load to the bone, supporting the mandible, and rehabilitating the occlusion with fixed dentition. In addition, finite element analyses were performed to calculate mandibular and implant stress under load application to verify the design functionality.

## 2. Material and Method

**Figure 1:**
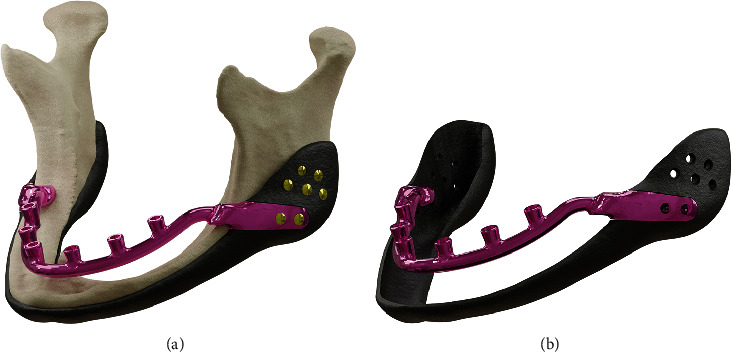
Implant assembly (a) including mandible geometry and (b) excluding mandible geometry.

Patients with extensive atrophy in the basal bone and interforaminal area who are classified as having Class V or VI mandible resorption, according to the Cawood classification, are the target patients for this approach [9]. A geometrical model of the resorbed mandible and implant idea based on three-dimensional computed tomography (CT) scan DICOM^1^ images (0.5 mm slice thickness) of a case with the previously indicated background was created. The implant comprises two distinct components: the mandibular cover and the horseshoe-shaped device which sits within the mouth and above the gum to hold the abutments. After fitting into the cover, the horseshoe component is put in place using two screws on each side (Figures 1(a) and 1(b)). A submandibular incision could be used to register the mandibular cover, and two minor intraoral incisions on the retromolar mucosa could be used to fit the horseshoe component to the cover. Given the personalized nature of this idea, the most significant anatomical issue when building the horseshoe structure is establishing a Class 1 occlusion. The cover should also have an insertion route that, following registration, results in the least amount of undercut.

The primary aim of this design was to reduce the stress experienced by the interforaminal and basal bones, hence reducing the risk of bone fractures and nerve irritation, as is the case when using traditional implants [10]. In order to accomplish this, the horseshoe was supported by a cover in the ramus and angle regions. To avoid touching the gingiva and upsetting the alveolar nerve during the horseshoe's deflection under load application, a 1 mm gap between the gingiva and the horseshoe was taken into consideration. Five screw positions were placed on each side to be used at the surgeon's discretion. Due to the weakening effect of the screw locations on the bone, no screw locations were considered on the mandibular body.

Two distinct finite element models in ABAQUS (Simulia, Rhode Island, United States) with varying numbers of screws (i.e., three or five on each side) were developed in order to investigate the impact of the number of screws on the stress distribution around the screw locations. Isotropic Ti6Al4V material properties were used for the screws and implant, whereas cortical bone properties were applied to the jaw (mechanical properties for the materials have been listed in Table 1) [11, 12]. For contact zones such as bone with screws and implants, as well as interface areas between the horseshoe and the cover, standard surface-to-surface (master–slave) contact with hard contact normal behavior and penalty tangential behavior with a coefficient of friction equal to 0.8 were defined. In contrast, a tie constraint was utilised for the interface regions between the screws and the implant. The temporomandibular joint and coronoid area were defined as fixed boundary conditions.

**Figure 2:**
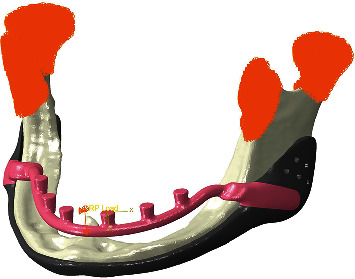
Boundary condition and 1 mm deflection at the reference point in the *z*-axis of posts.

**Figure 3:**
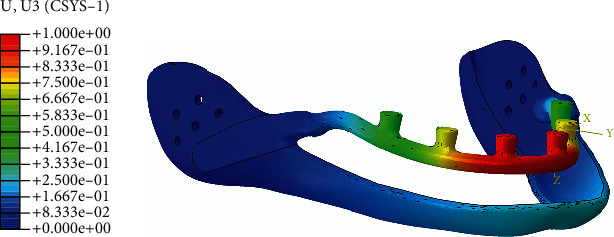
Horseshoe vertical deflection under load application (millimeter).

**Figure 4:**
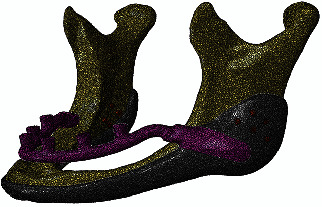
3D mesh of parts.

The reference point in the center of the anterior posts, which has an MPC (multiple point constraint) beam constraint with the horseshoe section to replicate the essential gingiva contact, was given a 1 mm vertical deflection along the longitudinal direction of the posts (Figures 2 and 3). All components were meshed with a three-dimensional four-node tetrahedral (C3D4) element with a 0.5 mm triangular edge length (Figure 4).

## 3. Results

**Figure 5:**
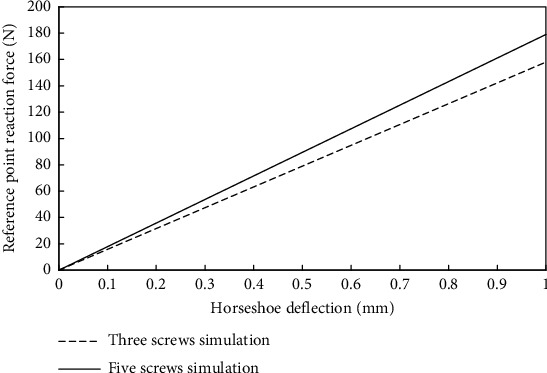
Reaction force at the reference point in terms of horseshoe deflection.

For each simulation, the 3D color contours of Von Mises stress distribution were extracted for the implant's individual components and the mandible, as well as the nodal reaction force (only in the *Z* direction of the postcoordinate system) at the loading reference point as a function of the vertical displacement of the horseshoe. The reaction force represents the magnitude of load that the patient must apply to cause contact between the horseshoe and gingiva, causing potential pain. At 1 mm deflection of the mandibular horseshoe, the chewing reaction force was shown to be under 200 N (Figure 5).

**Figure 6:**
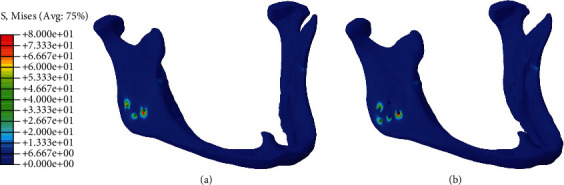
Von Mises stress distribution on mandible for fixations with (a) three and (b) five screws (megapascal).

**Figure 7:**
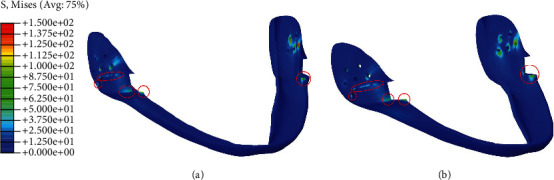
Von Mises stress distribution on cover for fixations with (a) three and (b) five screws (megapascal).

**Figure 8:**
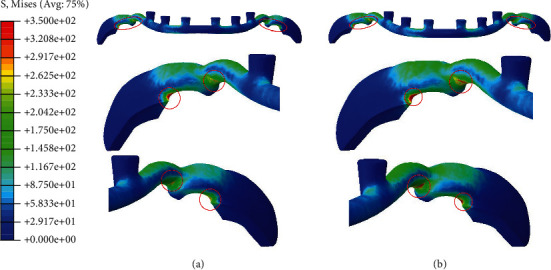
Von Mises stress distribution on horseshoe for fixations with (a) three and (b) five screws (megapascal).

Minimal stress was observed in the mandible during either simulation, with the exception of the area near the screw locations (Figures 6(a) and 6(b)). In the cover, however, contours illustrated that stress was present (approximately 20 MPa) in the body, besides the stress concentration at the screw locations and contact interfaces of the horseshoe (Figures 7(a) and 7(b)). The horseshoe and its connectors experience bending stress resulting from the deflection of the horseshoe; additionally, they are affected by the contact stress of the cover which makes them the most important section of design with stress values approximately equal to 200 MPa and stress concentrations approximately equal to 350 MPa in the vicinity of sharp edges (Figures 8(a) and 8(b)). Also, on the corresponding contact area in the cover, the stress was measured to be between 30 and 40 MPa, with the maximum stress concentrations in the vicinity of sharp edges being equal to 100 MPa in the simulation with the three screws (Figures 7(a) and 7(b)).

## 4. Discussion

Stress contours suggest that the effective load transfer within the ramus, from the horseshoe to the cover and the screw locations, would minimize the risk of nerve damage and mandibular fracture. Additionally, increasing the number of screws further reduced the stress on the bone, potentially reducing the risk of long-term screw loosening. However, extending the cover over the ramus to achieve more screw locations necessitates extensive soft tissue dissection and potentially using Trocar screw systems, which could lead to undesirable facial scarring.

Despite the fact that the experienced load on the mandibular body is low, there would be a minimum risk of further alveolar ridge resorption due to the fact that this design is proposed for patients who fall in the last group of the Cawood resorption classification, having undergone the maximum alveolar ridge resorption with a height less than 10 mm [9]. Studies have also indicated that the maximum resorption in either gender is about 50% of the height of the ridge on average, which corresponds to the Class V and VI Cawood classifications [13, 14]. Given the fact that candidate patients for this concept already have the highest possible residual ridge resorption, the possibility of further bone loss due to stress shielding would be significantly low.

Compared to endosteal implants and overdentures, considerations like patients' bone condition, drilling protocol selection, fixture material, length, and osteointegration would not be a concern in cases treated using the proposed design [15–18]. The abutments could be placed on the horseshoe bar without the need for a fixture, which allows patients to use a denture after the incisions are healed. Other solutions, like subperiosteal implants, endosteal implants, and overdentures, would need distributed bone support fixations which could cause further bone loss caused by stress shielding around the fixture and screws, potentially leading to loosening and failure [19, 20]. However, in this concept, all screw fixations are located in the ramus region, which would have reduced resorption risk compared to the basal bone and interforaminal area.

The concept was proposed for patients with a fully edentulous mandible who are not suitable for standard and subperiosteal implant surgery due to insufficient bone volume and height, which could result in further bone resorption or fracture. The current gold standard treatment involves a two-stage approach: bone graft augmentation followed by implant placement, which would demand a prolonged treatment period. The proposed concept, however, would enable patients to use dentures upon incision healing, significantly reducing treatment duration. Additionally, eliminating the need for bone grafting and implant fixtures would result in a substantial decrease in overall treatment costs. Nevertheless, employing this concept requires careful preoperative planning to ensure successful postoperative results. Diabetes and addiction would highly increase the risk of implant exposure [21]. Poor oral hygiene could significantly increase the risk of saliva and food particle leakage in the wound, followed by infection [22]. Regarding the surgical procedure, it would be worth mentioning that horseshoe fitting into the cover would be easier when both connectors are placed in their locations simultaneously rather than one at a time. Furthermore, since candidates for this approach are usually elderly patients with skin wrinkles, the submandibular incision for the cover could be positioned along these skin features to minimize the appearance of the scar tissue.

In the normal daily activities of healthy individuals, the bite force could be in any direction and at various amplitudes [23]. The horseshoe was designed to have a 1 mm gap from the gingiva to avoid any nerve disturbances. In the study, only the normal direction of the bite force was considered, and the boundary condition was applied only along the *z*-axis of posts to isolate the vertical deflection of the horseshoe during bending, which causes the horseshoe to contact the gingiva (Figures 2 and 3). The bite force value may vary from anterior teeth to posterior teeth [24], and as such, the worst-case scenario was considered in the present study, where all loads are applied to the anterior structures, resulting in the highest bending moment and its subsequent deflection.

During the chewing process, in healthy individuals, the bite force value may range from 70 to 900 N [24]. However, patients suffering from the severely atrophic mandibular ridge are not able to exert a force this great. With the maximum reaction force around 200 N in the worst-case scenario, it could be concluded that no contact with the gingiva is made. Consequently, nerve disturbance may not occur due to the deflection of the horseshoe when patients with severely atrophic ridges apply such chewing loads.

**Figure 9:**
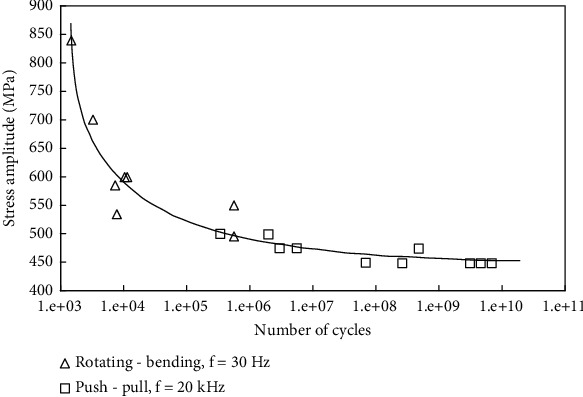
SN diagram of Ti6Al4V [25].

The analysis results revealed stress concentrations at the contact areas of the horseshoe and the cover. Postproduction surface modification processes such as polishing and anodizing could be used to improve fatigue life considerations due to these stress concentration points. Thus, the most critical section with a possible risk of fatigue would be the interface area between the horseshoe and its connectors, with stress measured up to 350 MPa. This is significantly lower than the Ti6Al4V yield stress (1100 MPa) as well as its unlimited fatigue life threshold. According to the Ti6Al4V SN diagram (Figure 9), for stress values around 350 MPa, the predicted life cycle exceeds 10^10^ [25]. Considering the 1050 chewing cycles per day for humans, the estimated life cycle for the implant under this loading condition would be concluded to be infinite [26].

## 5. Conclusion

This study is aimed at presenting a new design concept for patient-specific implants for the treatment of severely atrophic mandibles that can alleviate the possible complications of conventional treatment options. The finite element analysis results showed that this concept would be functional as the minimum load would be transmitted to the mandibular ridge, minimizing the risk of bone fracture and nerve disturbance. However, more bone screw fixations are needed to reach the minimum amount of stress on the screw region and the risks of long-term screw loosening. The finite element results are a valuable preclinical assessment of this design concept, but they would not be a sufficient criterion to prove the clinical success of this concept. Clinical trials and case series with multiyear follow-ups are needed for a detailed analysis of the suitability of this concept.

## Figures and Tables

**Table 1:** Mechanical properties of different parts of the model.

**Material**	**Density (g/cm** ^**3**^ **)**	**Elastic modulus (MPa)**	**Yield strength (MPa)**	**Poisson's ratio (v)**
Mandible	1.8	18,300	—	0.3
Ti6Al4V	4.43	110,000	1100	0.3

## Data Availability

The datasets used and/or analyzed during the current study including ABAQUS simulation files are available from the corresponding author on reasonable request.

## Endnotes

^1^Digital Imaging and Communications in Medicine
